# Association of *MIR137* With Symptom Severity and Cognitive Functioning in Belarusian Schizophrenia Patients

**DOI:** 10.3389/fpsyt.2018.00295

**Published:** 2018-07-05

**Authors:** Hanna Kandratsenka, Anastasiya Nestsiarovich, Inna Goloenko, Nina Danilenko, Anna Makarevich, Victor Obyedkov, Oleg Davydenko, Napoleon Waszkiewicz

**Affiliations:** ^1^Laboratory of Cytoplasmic Inheritance, Institute of Genetics and Cytology, National Academy of Sciences of the Republic of Belarus, Minsk, Belarus; ^2^Department of Internal Medicine, Center for Global Health, University of New Mexico, Albuquerque, NM, United States; ^3^Department of Psychiatry and Medical Psychology, Belarusian State Medical University, Minsk, Belarus; ^4^Department of Psychiatry, Medical University of Białystok, Białystok, Poland

**Keywords:** schizophrenia, gene, polymorphism, sex, *miR137*, Rs1625579

## Abstract

MicroRNA-137 (miRNA-137; miR-137) is one of the important post-transcriptional regulators of the nervous system development, and its *MIR137* gene rs1625579 polymorphism was reported to be a potential regulator for schizophrenia susceptibility. However, schizophrenia characteristics controlled by *MIR137* rs1625579 polymorphism are still insufficiently understood. There were 3 groups included in the study: (a) subjects with diagnosis of schizophrenia (*n* = 150; 81-females, 69-males), (b) mentally healthy people (control group; *n* = 102; 66-females, 36-males) and (c) Belarusian indigenous male group (*n* = 295). Associations of rs1625579 with schizophrenia, symptom's severity and cognitive performance [by using Positive and Negative Syndrome Scale (PANSS) and Wisconsin Card Sorting Test (WCST), respectively] were studied, when compared to controls. Allele and genotype frequencies were investigated in Belarusian indigenous males. Rs1625579 displayed no association with schizophrenia in Belarusian population. Significant “symptom severity-genotype” interactions were revealed for schizophrenia patients. Patients with T/G genotype displayed lower severity of positive symptoms and general psychopathology compared to homozygous subjects. T/T genotype was associated with the highest symptom's severity. The negative symptom scores and the total PANSS-score were significantly higher in females carrying genotype T/T vs. T/G+G/G; no significant gene-phenotype associations were found in males. WCST parameters did not show any association with rs1625579 polymorphism. *MIR137* rs1625579 polymorphism might be an important sex-dependent factor influencing severity of schizophrenia psychopathological manifestations. These findings also contribute to the knowledge on candidate gene effects on characteristics related to schizophrenia phenotype. As miR 137 is considered to be cancer therapeutic target, miR-137 may also explain the lower incidence of cancer in schizophrenia patients. Further studies with larger sample size are needed to confirm these novel findings.

## Introduction

According to the current concepts, mental disorders are the result of the complex interactions between genetic determinants and epigenetic factors. Molecular mechanisms of epigenetic modifications include histone modifications of chromatin, DNA methylation, RNA editing, as well as the effect of non-coding RNA molecules such as small interfering RNA (siRNA) and microRNA (miRNA) (1). MicroRNAs (miRNAs) are important post-transcriptional regulators of gene activity, the changes of its expression and functional status may have various consequences. MiRNAs were recently reported to be related to rheumatoid arthritis, systemic lupus erythematosus, diabetes, multiple sclerosis, asthma, psoriasis, inflammatory bowel diseases, dermatological disorders, psychiatric and neurological disorders (2). The aberrant expression of miRNAs was shown to be associated with schizophrenia, autism, Down syndrome, Rett syndrome, Fragile X syndrome, and depression (2, 3). A number of severe mental disorders, particularly schizophrenia, are characterized by presence of comorbid diseases. Such conditions may be partly explained by the variations in miRNAs functioning—one of the key elements of the gene set regulation that might be intrinsically related to the common etiology of several diseases.

The Genome-Wide Association Studies (GWAS) have shown that *MIR137* gene may be considered as a strong candidate to be included to the existing list of 108 gene loci of schizophrenia susceptability (4–6). Some lines of evidence show that individuals suffering from mental disorders, in particular schizophrenia, displayed altered *MIR137* expression as well as its target genes (6). MiRNA-137 is known to regulate the expression of ca. 1 900 other genes including several genes independently associated with schizophrenia (7). Such well-known candidate genes targeted by miR-137 include *BDNF, ZNF804A, TCF4, CACNA1C, CSMD*, and *C10ORF26* (8, 9). The product of *MIR137* gene, microRNA-137 (miR-137), may be involved in pathophysiology of schizophrenia by affecting synaptogenesis, neurogenesis in adults, synaptic plasticity as well as many other aspects of the central nervous system development (6, 9, 10). The mice models confirmed the effect of miR-137 on neurogenesis, proliferation and maturation of the neural stem cells (11). The level of miR-137 in adult brain tissue is about 100 times higher than in embryonic one, thus indicating that *MIR137* gene expression increases during brain development and neuron differentiation (8). Several brain regions including subcortical (caudate nucleus, putamen), cortical (orbito-frontal cortex) areas and synapto-dendritic neuron compartments are enriched with *MIR137* (8).

Recently, special attention was drawn to the study of *MIR137* association with the structural brain abnormalities and the patterns of functional activation of certain brain areas. Several lines of evidence displayed controversial results, one of them found that schizophrenia patients with T/T genotype of *MIR137* rs1625579 polymorphism, but not healthy subjects, had abnormal size of subcortical structures (increased lateral ventricles and reduced hippocampus); other authors failed to confirm this association neither in patients nor in the healthy subjects (8, 12). Li and Su (13) reported the data on rs1625579 polymorphism association with the size of amygdala, caudate nucleus, hippocampus, shell and the total brain volume in 299 Chinese and 5775 Caucasian subjects. In Chinese population, significant associations were found for hippocampus size only, with smaller size observed in T allele carriers (13).

**Figure 1:**
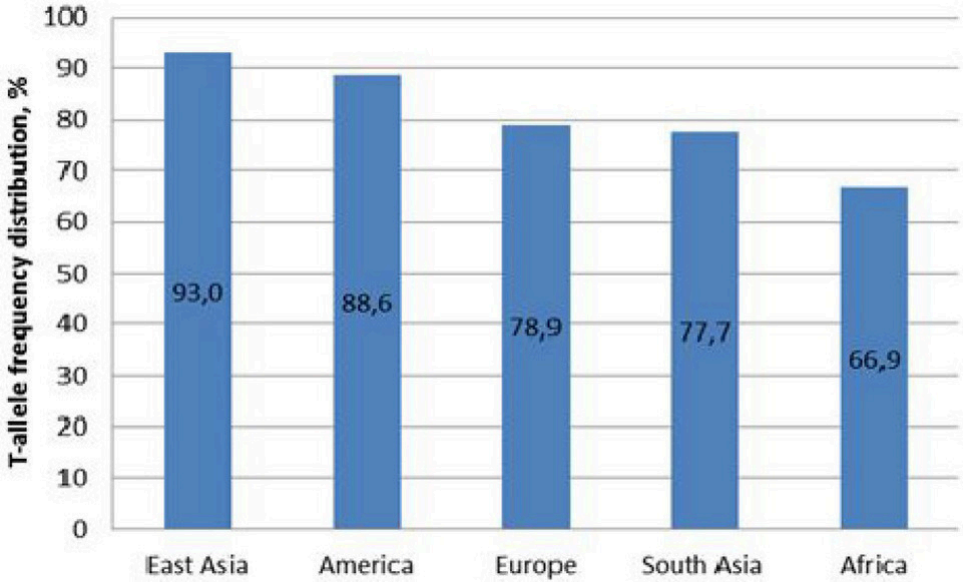
T-allele frequency of *MIR137* rs1625579 polymorphism in world populations. Comment: East Asia is represented with Chinese, Japanese and Vietnamese populations; America with Columbian, US (subjects of Mexican origin living in Los Angeles), Peruvian, and Puerto-Rican populations; Europe with Finnish, British, Spanish, Italian populations and subjects of European origin living in Utah state; South Asia with Bangladesh, Pakistani populations and subjects living in US and UK originating from India and Sri Lanka; Africa with Kenyan, Nigerian, Gambian, Sierra-Leonean populations and subjects of African origin living in US and Barbados.

Currently, the studies of *MIR137* rs1625579 association with susceptibility to schizophrenia hold the leading position, nevertheless the number of data points to the contradictory results for different ethnic groups (Table 1, Figure 1). GWAS studies have shown the association between rs1625579 (*p* = 1.6 × 10–11) SNP with schizophrenia, however, it is still difficult to explain the functional significance of the SNP localized in the intron region of *MIR137* primary transcript (6, 12). The major T-allele of rs1625579 polymorphism was identified as a risk factor for schizophrenia. Postmortem studies showed that subjects with T/T genotype have reduced level of *MIR137* expression compared to other genotypes carriers that enabled to suggest the impact of this locus on *MIR137* gene expression (1, 6, 12).

**Table 1:** Association studies of *MIR137* rs1625579 polymorphism with risk of schizophrenia.

**Source**	**Population**	**Schizophrenia patients**				**Healthy controls**				***P*-value**
		***N***	**Genotype frequencies (%)**			***N***	**Genotype frequencies (%)**			
			**T/T**	**G/T**	**G/G**		**T/T**	**G/T**	**G/G**	
Sun et al. (14)	China	589	86.9	12.2	0.9	622	86.8	12.7	0.5	*p* > 0.05
Kuswanto et al. (8)	Singapore	84	89.2	10.8		63	88.9	11.1		*p* > 0.05
Yuan et al. (15)	China	506	87.9	11.7	0.4	522	89.6	10.4	0	*p* > 0.05
Wang et al. (16)	China	300	89.7	10.3	0	300	88.3	11.7	0	*p* > 0.05
Guan et al. (10)	China	1429	77.7	20.8	1.5	1570	74	23.6	2.4	***p*** = **0.023**
Van Erp et al. (11)	US	48	81.25	18.75	0	63	73.02	23.81	3.17	*p* > 0.05
Hommers et al. (5)	GWAS, 2014	Caucasians n (patients) = 33.332 n (controls) = 27.888								*p* < 0.05
	GWAS, 2013	Sweden samples: n (patients) = 5.001, n (controls) = 6.243; Repeated study 1 (Caucasians): n (patients) = 8.832, n (controls) = 12.067; Repeated study 2 (Caucasians): n (patients) = 7.413, n (controls) = 19.762								*p* < 0.05
	GWAS, 2011	Caucasians n (patients) = 9.394 n (controls) = 12.462 Repeated study n (patients) = 8.432 n (controls) = 21.397								*p* < 0.05

*GWAS, genome wide association study. Bold values indicate the mean statistical significance or % value*.

British scientists made an attempt to clarify the functional significance of rs1625579 locus for *MIR137* gene. It was shown that rs1625579 is in linkage disequilibrium with the rs2660304 polymorphism located within an internal promoter of (Imir137) *MIR137* gene. By using the reporter gene construct it was showed that rs2660304 alleles affect transcriptional activity of the Imir137 promoter of *MIR137* gene. As rs1625579 (a risk factor for schizophrenia), is in linkage disequilibrium with rs2660304, a possible functional role of rs1625579 as a factor regulating *MIR137* expression by means of rs2660304 polymorphism was suggested (6).

The data pool regarding *MIR137* effect on schizophrenia liability is increasing; however, the understanding of schizophrenia clinical features regulated by this gene is still insufficient. In the present publication, we analyzed the association of *MIR137* rs1625579 gene polymorphism with susceptibility to schizophrenia in Belarusian population, as well as its relationship with symptom severity and cognitive functioning.

## Materials and methods

### Object of the study

There were 3 groups (mean age ± SD [46.8 ± 9.4], min-max [24–65]) included in the study: (a) subjects with diagnosis of schizophrenia according to ICD-10 criteria (main group), (b) mentally healthy people (control group), (c) Belarusian indigenous group. Schizophrenia patients consisted of 150 individuals (81 females and 69 males), control group consisted of 102 people (66 females and 36 males), and Belarusian indigenous group contained 295 males whose ancestors lived in Belarus for at least 3 generations. There were no age differences between groups. Recruitment of subjects with schizophrenia was carried out by psychiatrists of the Republican Research and Practice Center for Mental Health (RPCMH, Minsk) among the in-patients undergoing the course of treatment. The study was approved by the ethic committee of the RPCMH, all participants gave signed written informed consent and biological samples (capillary blood) were taken afterwards.

Clinical assessment of patients was performed by psychiatrists of RPCMH at the stage of schizophrenia “clinical outcome” which reflects the period of maximal symptoms consolidation and appears 12–15 years after the onset of psychotic symptoms (17). The following inclusion criteria were applied: age 18–65 years, disease duration over 12 years, no evidence of regular neuroleptics intake during 1–3 weeks prior to hospitalization, absence of any comorbid psychiatric disorders (except of nicotine dependence), absence of severe somatic states and neurological disturbances. The control group consisted of healthy individuals. These subjects were presented by volunteers of different socio-demographic groups (students, workers of medical and research institutions).

To increase the statistical power, a sample of Belarusian indigenous population containing 295 males whose ancestors lived in Belarus for at least 3 generations was included into this study. The biological material of the participants was obtained from the population DNA bank previously created in the Laboratory of Cytoplasmic Inheritance. Some details of all the samples are provided in Table 2.

**Table 2:** Gender characteristic in patients and controls.

**Characters**	**Patients**	**Controls**	**P^1^**
***N***	150	102	–
**Gender**			0.118
Female	54.0%	64.7%	
Male	46.0%	35.3%	

*P^1^, Comparison between the patient group and control group using Chi-square method; SD, standard deviation*.

### Clinical measurements

Patients were examined during the period of acute schizophrenia exacerbation (“relapse”). Majority of symptoms were measured during the first 3 days of hospitalization once a patient was able to establish the contact with investigator. To minimize the impact of the maintenance neuroleptic therapy on the disease course and relapse symptomatic structure, only patients who were not regularly taking antipsychotics during 1–3 weeks prior to the current hospitalization were selected for examination. This information was obtained from patient's caregivers, as well as from medical documentation (including the referrals).

Symptom severity was assessed by means of the semi-structured clinical interview and the psychometric scale PANSS (Positive and Negative Syndrome Scale) which consists of 33 items, rated from 1 to 7 scores each. All the items are arranged into four sub-scales reflecting the severity of positive symptoms, negative symptoms, general psychopathology and additional symptoms (18).

The cognitive functioning was evaluated by means of Wisconsin Card Sorting Test (WCST)—a widely used tool to assess the working memory, attention, abstract thinking and executive functioning (19). During the test it is presented a series of cards on the computer monitor with figures differing by either color, number or shape. The patient is asked to sort each of the 128 cards into four categories based on a single currently active criteria which changes from time to time. The test is considered to measure the ability of a testee to identify and follow the classification algorithm, and to productively “switch” once the rules are changed. We evaluated the following test variables: (1) perseverative errors which reflect the problems in switching from one sorting rule to another; (2) non-perseverative errors which are associated with absent-mindedness of attention; (3) total of all errors which reflects a functional status of cognitive flexibility and attention of the examinee; (4) total amount of categories completed which reflects the cognitive processing success during the test; (5) total amount of cards to complete the first category which reflects the efficiency/speed of identifying the first classification algorithm. The most informative variable for executive functioning is “perseverative errors.”

### Genotyping

Genomic DNA was extracted from peripheral blood leukocytes using the phenol-chloroform extraction (20). Genotyping of rs1625579 *MIR137* was performed using TaqMan assay. The primers and probes used for PCR amplification were designed by the Beacon Designer 7.91. software (primers: forward-5′-CCCTAGTTGAAAATATTTC-3′ and revers-5′-GGGTCACTATTATTTAACA-3′/probes: FAM-5′-TGTTAATCACAATTACATCAACTCAG-3′; HEX-5′-TGTTAATCACAATTAAATCAACTCAG-3′). PCR amplification was performed in a 25-μl reaction volume, which contained 2X Mix (Primetech, Minsk, Belarus: 0.04 u/μ of Diamant FAST DNA polymerase 10X Buffer ≪FAST≫ containing 4.5 mM MgCl2, 0.2 mM of each dNTP), 0.3 pM of each primer and probe, and 30–40 ng of genomic DNA. The conditions used for thermal cycling included initial denaturation at 95°C for 2 min, 10 cycles without fluorescence detection at 95°C for 15 s, at 58°C for 30 s and at 72°C for 30 s, and a final elongation at 72°C for 40 s after that 35 cycles with the same mode but with fluorescence detection. The described thermal cycling was performed with the Real-Time Stratagene Mx 3005P (Agilent Technologies, Santa Clara, California). Allele designations for *MIR137* rs1625579 are according to the NCBI reference sequences and the dbSNP database (https://www.ncbi.nlm.nih.gov/projects/SNP/snp_ref.cgi?rs=1625579).

### Statistical analysis

The association of the studied locus polymorphism with schizophrenia symptom severity and cognitive functioning was tested with SPSS software Version 22.0. The two-tailed estimation of significance used in the analysis was defined at *P* < 0.05. The Hardy–Weinberg (HWE) equilibrium was verified for patients and controls. The presence of HWE was tested by chi-square test (χ^2^) for goodness of fit. Chi-square method (Pearson's X2) was used to evaluate rs1625579 locus for the risk of schizophrenia. Association of rs1625579 variants with variables related to schizophrenia symptom severity and cognitive functioning was analyzed using non-parametric Kruskal-Wallis *H*-Test and Mann-Whitney *U*-test. Association of rs1625579 polymorphism with symptom severity and sex was tested using two-way ANOVA.

## Results

Analysis of genotype and allele distribution of *MIR137* rs1625579 polymorphism in both compared groups has shown the T allele prevalence and has not revealed any significant differences between patients and controls (Table 3). The distribution of Allele frequencies of the locus studied in the control group corresponded to that known for European populations (“1000 genomes” database) (Electronic source: http://browser.1000genomes.org/Homo_sapiens/Variation/Population?db=core;r=1:98502434-98503434;v=rs1625579;vdb=variation;vf=1146801#373401_tablePanel). The allele frequencies were consistent with Hardy–Weinberg equilibrium for patients and controls. Allele frequencies analysis in a sample of Belarusian indigenous population has demonstrated high T allele total frequency: T allele −75.9%; G allele −24.1%. The detailed data on the genotype frequencies in Belarusian indigenous population compared to the world-wide populations obtained within the “1000 genomes” project are presented in Table 4.

**Table 3:** Allele frequencies distribution of *MIR137* rs1625579 polymorphism among schizophrenia patients and healthy controls.

**Allele**	**Patient group**	**Control group**	***P*-level; α = 0.05**
T	77%	74%	*p* = 0.459
G	23%	26%	

**Table 4:** Genotype distribution of *MIR137* rs1625579 polymorphism in world populations.

**Populations**	**Genotypes**					
	**T/T**		**T/G**		**G/G**	
	***n***	**%**	***n***	**%**	***n***	**%**
Belarusian indigenous population, *n* = 295	178	**60.3**	92	**31.2**	25	**8.5**
East Asia, *n* = 504	437	**86.7**	63	**12.5**	4	**0.8**
America, *n* = 347	274	**79.0**	67	**19.3**	6	**1.7**
Europe, *n* = 503	314	**62.4**	166	**33.0**	23	**4.6**
South Asia, *n* = 489	288	**58.9**	184	**37.6**	17	**3.5**
Africa, *n* = 661	306	**46.3**	273	**41.3**	82	**12.4**

*Structure of presented populations is identical to that in Figure 1. Bold values indicate the mean statistical significance or % value*.

A comparative analysis of the genotype frequencies and combinations (T/T vs. T/G+G/G) of rs1625579 polymorphism has not revealed any significant differences between schizophrenia patients and healthy controls (Table 5). The non-significant increase of T/T genotype frequency was observed in patient group compared to the control group, and this trend persisted when sexual differences were taken into account (patient female T/T – 65.4% vs. control female T/T – 54.5%; patient male T/T – 55.1% vs. control male T/T – 50%). However, the overall genotype frequencies distribution did not differ significantly between the comparison groups, both in males and females (*p* = 0.480 and *p* = 0.362, respectively). Also, we didn't find any differences in allele and genotype distribution between male patients and a sample of Belarusian indigenous population (*p* = 0.579).

**Table 5:** Genotype frequencies distribution of *MIR137* rs1625579 polymorphism in schizophrenia patients and healthy controls.

**Genotype**	**Patient group, % (subjects)**	**Control group, % (subjects)**	***P*****-level;** α = **0.05**		**OR (95% CI)**
T/T	60.7% (91)	52.9% (54)	0.244	*p* = 0.294 X^2^ = 2.445	1.37 (0.82–2.28)
T/G	32.7% (49)	42.2% (43)	0.143		0.67 (0.4–1.12)
G/G	6.7% (10)	4.9% (5)	0.787		1.39 (0.46–4.18)
Total number of subjects	150	102	

*OR, odds ratio; CI, confidence interval*.

**Figure 2:**
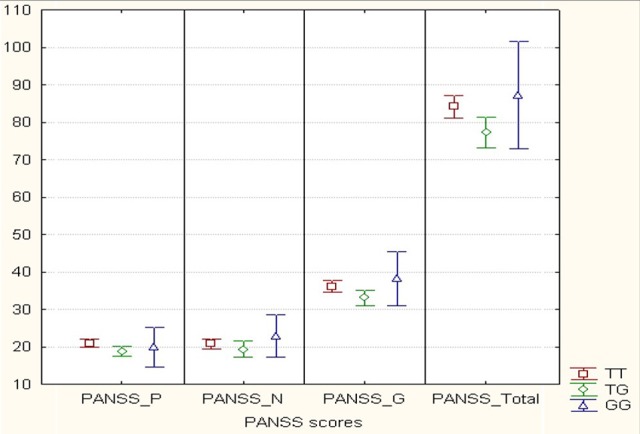
Mean values (score) of positive, negative and general psychopathology subscales of PANSS according to *MIR137* rs1625579 polymorphism in schizophrenia patients. Comment: Graphic elements reflect Mean + 0.95 CI; X-axis is expressed in score values and Y-axis contains four totals of positive (PANSS_P), negative (PANSS_N), general psychopathology (PANSS_G) items and total index for all subscales (PANSS_Total). Sample volume: TT - 91 subjects, TG - 49 subjects, GG - 10 subjects. *P*-value < 0.05 for PANSS_P, PANSS_G and PANSS_Total.

We analyzed *MIR137* rs1625579 polymorphism association with the schizophrenia symptom severity using non-parametric Kruskal-Wallis-H-test for genotypes, and Mann-Whitney *U*-test for genotype combinations in 150 schizophrenia patients. The average scores for the different PANSS subscales (positive symptoms, negative symptoms, general psychopathology) and the general index for all subscales of psychometric PANSS are presented in Figure 2. These data demonstrate that rs1625579 polymorphism is significantly associated with positive symptoms and general psychopathology scores as well as with the total symptomatology score in patients with schizophrenia. To achieve a higher resolution of the observed trend, we analyzed the genotype and genotype combination associations with each of the symptom complex (Table 6). The results obtained show the presence of a “heterozygote effect” of rs1625579, which implies that in subjects possessing heterozygous TG genotype, the severity of symptom (PANSS_P, PANSS_G, PANSS_Total) is significantly lower when compared to homozygous subjects. At the same time, T/T genotype is the most unfavorable for the abovementioned symptoms, syndromes and general psychopathological state of patients. It's worthy to note that statistical power of the studied sample was limited by a low number (10) of G/G homozygous subjects. It caused the simultaneous presence of significant differences between G+ (T/G+G/G) and G– (T/T) genotype combination (in this case G/G genotype plays a protective role) as well as between T/T+G/G and T/G genotype combinations (in this case G/G genotype has a negative effect upon symptoms severity) for PANSS_P and PANSS_Total indices. That's why a definite statement on G/G genotype effect on schizophrenia symptom severity in the studied sample seems to be difficult. Based on all, we can suppose that patients with T/T and possibly G/G genotypes are characterized with somewhat more severely expressed symptomatology compared to heterozygous subjects (*p* = 0.005).

**Table 6:** Significant association of *MIR137* rs1625579 polymorphism with symptom expression degree according to PANSS in schizophrenia patients.

**Subscales**	**Mean symptom expression degree, points**			**P-level, α = 0.05**
	**T/T (91 subjects)**	**T/G (49 subjects)**	**G/G (10 subjects)**	
**TOTAL INDEX OF POSITIVE SYMPTOMS**				
PANSS_P	**TT**	**TG**	**GG**	
	21.02 ±5.6	18.76 ± 4.52	20.00 ±7.42	**0.043**
	**TG**+**GG**	**TT**		
	18.97 ±5.07	21.02 ±5.6		**0.015**
	**TT**+**GG**	**TG**		
	20.92 ±5.77	18.76 ±4.52		**0.016**
**TOTAL INDEX OF GENERAL PSYCHOPATHOLOGICAL SYMPTOMATOLOGY**				
PANSS_G	**TT**	**TG**	**GG**	
	36.30 ± 7.24	33.14 ± 7.17	38.20 ± 10.14	**0.057**
	**TT**+**GG**	**TG**		
	36.49 ± 7.54	33.14 ± 7.17		**0.019**
**TOTAL INDEX OF ALL PANSS SYMPTOMS**				
PANSS_Total	**TT**	**TG**	**GG**	
	84.53 ± 13.99	77.31 ± 14.46	87.20 ± 20.05	**0.018**
	**TG**+**GG**	**TT**		
	78.98 ± 15.79	84.53 ± 13.99		**0.016**
	**TT**+**GG**	**TG**		
	84.79 ± 14.59	77.31 ± 14.46		**0.005**

*PANSS_P, variable reflecting positive symptom complex of PANSS; PANSS_N, variable reflecting all symptoms of general psychopathological subscale of PANSS (reflects schizophrenic disorder severity in general); PANSS_Total, variable reflecting all PANSS symptoms. Values are represented as Mean ± SD. Bold values indicate the mean statistical significance or % value*.

Another question that has arisen during our study was if the studied locus is related to the sex-dependent peculiarities of symptom expression. ANOVA analysis showed us the tendency in association of sex and rs1625579 *MIR137* only for PANSS_Negative (*F* = 3.02, *p* = 0.08). During the analysis of the association of rs1625579 with symptom severity in men and women separately we found that female patients with T/T genotype have significantly higher level of negative symptoms severity when compared to those with G-allele, as opposed to the male patient sample (Table 7). In male patients, the studied locus was significantly associated with one symptom only (“Poor attention”) however it is very likely that this correlation might most probably appear false-positive because of the small sampling size of patients with G/G genotype. Positive symptoms and a number of general symptoms have not shown statistically significant association with sex separately, and this fact may be explained either with small sampling size or with sexual specificity. Moreover, we found that rs1625579 is associated with negative symptoms severity in female patients only, thus supposing certain sex-dependent differences exist in the modes of rs1625579 impact on pathophysiological processes of symptomatic pattern development. In the whole schizophrenia group, carriers of (T/G) genotype of *MIR137* rs1625579 polymorphism demonstrated less severe positive symptoms and general psychopathology.

**Table 7:** *MIR137* rs1625579 polymorphism association with negative symptom expression degree according to PANSS in patients with schizophrenia.

**Subscales**	**Mean symptom expression degree, points**		**P-level, α = 0.05**
	**T/T**	**T/G+G/G**	
	Female patients		
	54 subjects	28 subjects	
PANSS_N	20.06 ± 6.91	17.00 ± 5.87	**0.043**
	Male patients		
	38 subjects	31 subjects	
PANSS_N	21.76 ± 6.37	22.55 ± 8.16	**0.659**

*Values are represented as Mean ± SD. Bold values indicate the mean statistical significance or % value*.

Analysis of Wisconsin Card Sorting Test (WCST) data obtained from 129 patients with schizophrenia has not revealed any association of rs1625579 polymorphism with the studied parameters (Table 8).

**Table 8:** *MIR137* rs1625579 polymorphism association with WCST variables in scizophrenia patients.

**WCST variable**	**Genotypes**		
	**T/T (83 subjects)**	**T/G (38 subjects)**	**G/G (8 subjects)**
Perseverative errors, %	17.8	19.1	16.4
Non-perseverative errors, %	28.3	32.3	16.0
Total of all errors, %	43.9	47.3	32.5
Total amount of categories completed (average number)	3.3	2.6	5.4
Total amount of cards to complete the first category (average number)	18.4	14.1	17.6

## Discussion

The analysis of the published literature has demonstrated a very ambiguous pattern of *MIR137* rs1625579 polymorphism associations with schizophrenia. According to the data presented in Table 1, three studies based on the GWAS that included Caucasian subjects have revealed a distinct association of rs1625579 locus with schizophrenia. However, a number of Chinese studies have not confirmed this association although it might be explained by the ethnical genetic differences. The Small sample size might also be a limiting factor for revealing the studied locus and schizophrenia association. In a recent meta-analysis on Chinese population studies which included 4 episode/control investigations with a total sample size of 2,847 patients and 3,018 healthy subjects, the authors have demonstrated significant association of the studied locus with schizophrenia for the following allelic models: T vs. G; T/T vs. G/T+G/G; T/T vs. G/G (21). On the other hand, another meta-analysis which included 11887 patients with schizophrenia and 16,660 healthy subjects has not revealed a studied locus and disease association in Asian populations, and it might be explained by the high heterogeneity in rs1625579 locus among continental populations (22).

Obtained data demonstrate high T-allele frequency of Belarusian indigenous population. T-allele is recognized as a major allele of *MIR137* rs1625579 polymorphism for all known populations. The frequency of Genotypes distribution in the studied Belarusian indigenous population complied with the one known for the European populations (Electronic source: http://browser.1000genomes.org/Homo_sapiens/Variation/Population?db=core;r=1:98502434-98503434;v=rs1625579;vdb=variation;vf=1146801#373401_tablePanel). The studied polymorphism demonstrates frequency heterogeneity across the world populations, with similar frequencies observed (a) in East Asian and American populations, (b) in European and South Asian populations. East Asian populations possessed maximal percentage of T/T genotype, as opposed to the European data. Since the world investigation data related to ambiguity of rs1625579 polymorphism and association with schizophrenia were mostly obtained from the East Asian populations, these ethnical differences were taken into account during the subsequent analysis and interpretation of this polymorphism association with schizophrenia risk.

Our analysis has revealed that *MIR137* rs1625579 polymorphism is associated with positive symptoms severity as well as with schizophrenia severity in general (PANSS). It might be mediated by dysregulation of a number of significant signal pathways determining nervous system development and function. For instance, bioinformatic analysis carried out by Wright et al. (23) has demonstrated that 1,144 genes represent potential targets of this miRNA, while 25 of them are considered to be schizophrenia risk factors. *MIR137* can influence on symptomatic indicators of patients and to interact with dopaminergic system. One of the miR-137 target genes is zinc finger protein 804A (*ZNF804A*) (8, 9). It participates in dopamine signaling pathways, and its target gene, catechol-*O*-methyltransferase (*COMT*), can directly and selectively degrade dopamine in prefrontal cortex synapses through methylation (24). When *COMT* expression is inhibited, the reduced dopamine degradation in prefrontal cortex synapses causes dopamine hyperactivity that could lead to symptom manifestation (24). The other *ZNF804A* target gene associated with the dopamine pathway is the dopamine receptor D2 (*DRD2*) gene (24). When the dopaminergic system in the mesolimbic pathway is hyperactive, the hyperactivation of the postsynaptic membrane D2 receptor can induce positive symptoms, such as hallucinations and delusions (24).

Based on the data with sex differences in the locus rs1625579, it is possible to assume that *MIR137* gene is involved in pathophysiological mechanisms leading to gender phenotypically distinct patterns of schizophrenia. Further investigation of the actual genes targeted by *MIR137* and the relevant signal pathways might be promising for new locus-specific medicinal agents development. A similar analysis carried out in patients with schizophrenia from Singapore has demonstrated a significant increase (*p* = 0.016) in negative symptomatology in T/T patients compared to the subjects with G/G or G/T genotypes (8). Data obtained by three independent Chinese investigation groups showed no association of the studied locus with scores for PANSS subscales (14, 16, 25). Possibly, these controversial results may be explained by ethnical heterogeneity as well as the limited sample sizes.

According to WCST data, obtained association of *MIR137* rs1625579 polymorphism with executive functioning have not been revealed but it could be false negative result due to small sample size. Kuswanto et al. (8) have analyzed rs1625579 polymorphism association with various neurocognitive domains, however, the significant association was revealed for executive functions only. This study has demonstrated that patients with schizophrenia homozygous by T allele have lower “attention” and “processing speed” cognitive variables. It's interesting that postmortem studies of brain tissue samples indicate that T allele may be associated with a decreased gene expression in dorsolateral region of the prefrontal cortex that is functionally involved into executive function in schizophrenia patients (10). Chinese research data published in 2014 have indicated that rs1625579 locus is associated with functional interaction between the dorsolateral region of the prefrontal cortex and hippocampal formation. Two hundred and ninety healthy subjects participated in this study. It was revealed that subjects homozygous by T-allele showed significant differences in abovementioned brain regions functional interactions compared to other genotypes. It was also demonstrated a strong association between the mentioned brain regions that predisposes to worse indices of working memory in T/G but not in T/T subjects. Therefore dorsolateral region of the prefrontal cortex is one of the most important brain regions involved in schizophrenia pathophysiology. Between this region and hippocamp connection formation, it was supposed that the studied polymorphism of *MIR137* gene may influence schizophrenia pathophysiology by affecting these brain regions development and interaction plasticity (9). Research group from US has revealed that TT genotype is associated with left dorsolateral prefrontal cortex hyperactivation compared to G/G and G/T genotypes both in patients with schizophrenia and the control group (11). Another study focused on genes that expression was altered by variations in *MIR137* expression. This list was cross-referenced with genome-wide schizophrenia association data to construct the individual polygenic scores. Increased polygenic risk within the empirically derived miR-137 regulated gene score was associated with significantly lower performance in intelligence quotient test, worse working memory and episodic memory. These effects were observed most clearly at a polygenic threshold of *P* = 0.05, although significant results were observed at all three thresholds analyzed. This result pointed out that *MIR137* gene associated with the risk of schizophrenia might also be related to its broader downstream genetic effects (7). It's worthy of note that we failed to reveal any association of the rs1625579 *MIR137* gene with Wisconsin Card Sorting Test (WCST) parameters of the number of cognitive processes that are predominantly controlled by prefrontal cortex. However, the data obtained in neurovisualization studies such as Functional Magnetic Resonance Imaging analysis etc. as well as studies based on different approaches to cognitive processes analysis have shown a functional significance of the mentioned locus for a number of cerebral structures, particularly prefrontal complex (5, 8).

In summary, the data obtained indicate that rs1625579 polymorphism of *MIR137* gene is associated with symptom severity in patients with schizophrenia; furthermore, sex-dependent differences of this association were revealed. The highest severity of symptoms was observed in patients carrying T/T genotype that is consistent with data on T/T genotype pathogenicity for nervous system functioning. Given the fact that a reduced adjusted incidence of cancer was noted in patients suffering from schizophrenia (by potential mechanisms such as excess of dopamine, enhanced natural killer cell activity, increased apoptosis, modulated cytochrome enzymes by antipsychotics that block mutagen activation, or vitamin D deficiency) (26) and miR-137 is considered as a cancer therapeutic target (27), our study could explain the lower incidence of cancer in schizophrenia compared to the general population. Taking into account high significance of *MIR137* for nervous system development and function, it's necessary to emphasize the importance of complex methodological approach to the studies of gene-phenotype interactions focusing on cognitive processes, that represent the basis of schizophrenia pathophysiology.

## Author contributions

HK, AN, IG, ND, AM, VO, OD, and NW designed the study and supervised the draft completion. HK, IG, ND, AM, and OD did genetic analyzes. HK, AN, and VO collected the cognitive and clinical data, managed the literature searches and performed the data analyses. HK, AN, and NW wrote the first draft of the manuscript. All authors contributed to and approved the final draft of the manuscript.

### Conflict of interest statement

The authors declare that the research was conducted in the absence of any commercial or financial relationships that could be construed as a potential conflict of interest.
